# Gene Flow and Genetic Diversity of a Broadcast-Spawning Coral in Northern Peripheral Populations

**DOI:** 10.1371/journal.pone.0011149

**Published:** 2010-06-16

**Authors:** Yuichi Nakajima, Akira Nishikawa, Akira Iguchi, Kazuhiko Sakai

**Affiliations:** Sesoko Station, Tropical Biosphere Research Center, University of the Ryukyus, Motobu, Okinawa, Japan; Northeastern University, United States of America

## Abstract

Recently, reef-building coral populations have been decreasing worldwide due to various disturbances. Population genetic studies are helpful for estimating the genetic connectivity among populations of marine sessile organisms with metapopulation structures such as corals. Moreover, the relationship between latitude and genetic diversity is informative when evaluating the fragility of populations. In this study, using highly variable markers, we examined the population genetics of the broadcast-spawning coral *Acropora digitifera* at 19 sites in seven regions along the 1,000 km long island chain of Nansei Islands, Japan. This area includes both subtropical and temperate habitats. Thus, the coral populations around the Nansei Islands in Japan are northern peripheral populations that would be subjected to environmental stresses different from those in tropical areas. The existence of high genetic connectivity across this large geographic area was suggested for all sites (*F*_ST_≤0.033) although small but significant genetic differentiation was detected among populations in geographically close sites and regions. In addition, *A. digitifera* appears to be distributed throughout the Nansei Islands without losing genetic diversity. Therefore, *A. digitifera* populations in the Nansei Islands may be able to recover relatively rapidly even when high disturbances of coral communities occur locally if populations on other reefs are properly maintained.

## Introduction

Coral reefs support the highest biological diversity of all marine ecosystems. Reef-building corals play an important role in structuring and maintaining coral reef ecosystems and in forming the framework of coral reefs. However, coral populations worldwide have been decreasing recently due to anthropogenic disturbances such as overfishing, sediment pollution, and nutrient influx and are also under threat from global warming and ocean acidification [1]–[3]. For the maintenance and recovery of coral populations, presenting reef conservation initiatives that consider population connectivity and the potential for corals to adapt to local environments is essential [4], [5].

Most coastal marine sessile organisms including corals have limited adult movement, so the relatively short, pelagic larval phase represents the primary opportunity for dispersal [6], [7]. Reef corals are able to move after settlement through asexual reproduction such as fragmentation [8]–[10] and polyp expulsion [11], but post-settlement dispersal distances are very limited. Asexually reproduced larvae may travel long distances, but this probably does not happen very often in corals [12]. Hence, the larval period plays an important role in the maintenance and habitat extension of coral populations. Although tracing the movement of marine larvae directly is generally difficult, many studies have estimated the extent of marine larval dispersal using genetic markers, e.g., crown-of-thorns starfish [13], French grunt [14], and blue mussel [15]. In corals, larval dispersal has been frequently estimated using allozymes (reviewed in van Oppen and Gates [4]), but previous analyses have mainly focused on connectivity over evolutionary, rather than ecological, timescales (using, e.g., Wright's *F*_ST_-based method). For more detailed examinations on the genetic connectivity of corals, analyses over ecological timescales using highly variable markers (e.g., microsatellites) are required. Recently, microsatellite markers have been developed for some corals (e.g., [16]–[18]) and have been used to investigate connectivity patterns between populations. Some such studies have shown that genetic differentiation was caused by oceanographic barriers (e.g., [19]). Relationships between genetic diversity and the geographic position of populations have also been surveyed, and decreases in genetic diversity have been detected in peripheral populations or with increasing latitude [20]–[23]. Using highly variable markers such as microsatellites would improve the management potential for target species [21].

Our target species *Acropora digitifera* is a broadcast-spawning coral that is widely distributed in Indo-Pacific coral reefs [24]. This species is one of the most common coral species in the Nansei Islands, Japan, which are home to many reefs supporting populations of various coral species [25]. Like other reef areas, coral populations in the Nansei Islands suffer from disturbances due to the above-mentioned anthropogenic factors [26]. In the Nansei Islands, *A. digitifera* also inhabits temperate areas, which represent the northern limit of this species' distribution [25]. Thus, the populations of *A. digitifera* around the Nansei Islands in Japan are the northern peripheral populations, which would be subjected to environmental stresses (e.g., temperature, light intensity) different from those in tropical areas.

**Figure 1:**
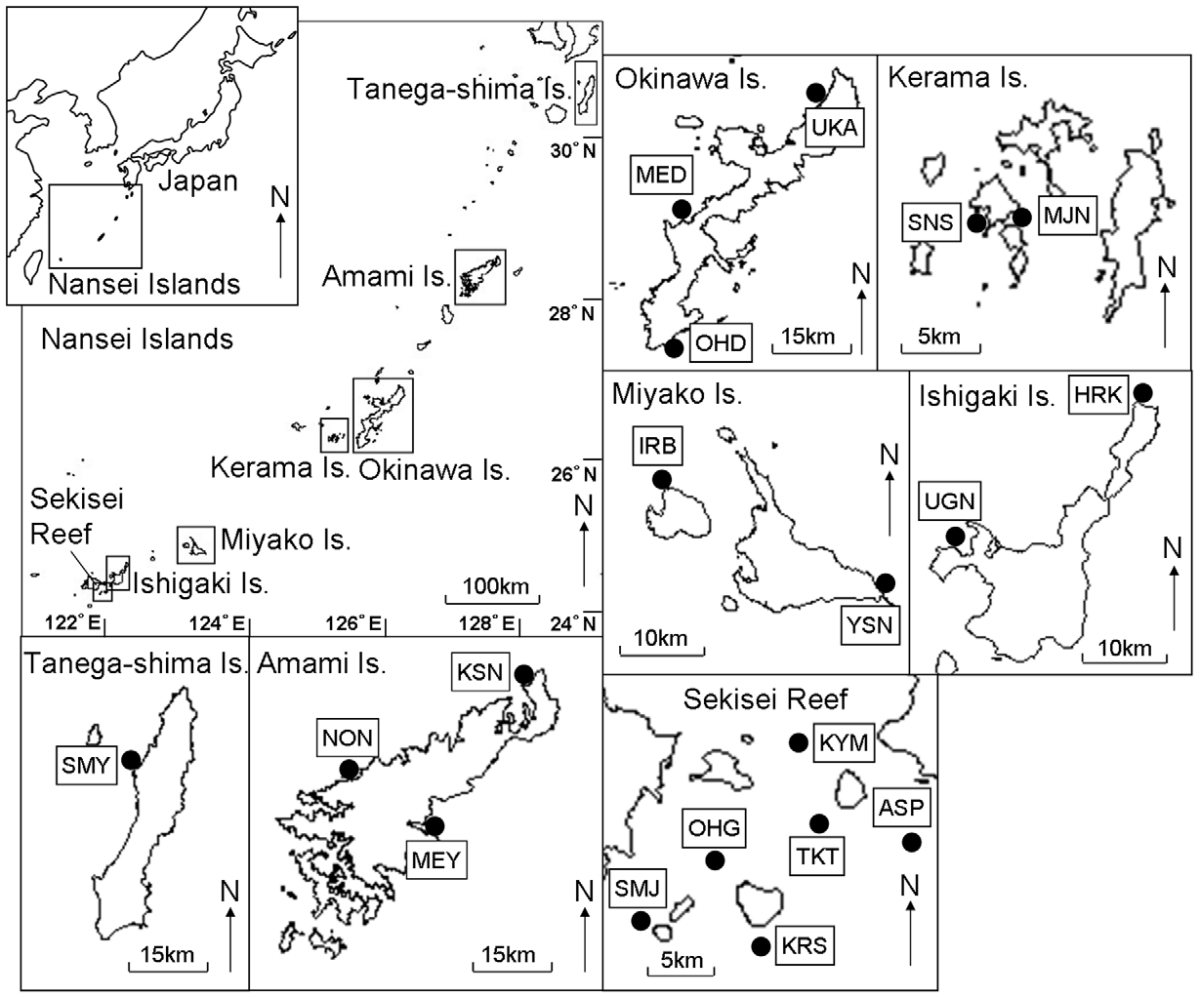
Map of the Nansei Islands showing the 19 sampling sites. •, Sampling sites: Sumiyoshi (SMY), Kusuno (KSN), Naon (NON), Maeyama (MEY), Ohdo (OHD), Maeda (MED), Uka (UKA), Majanohama (MJN), Sunashiro (SNS), Irabu (IRB), Yoshino (YSN), Uganzaki (UGN), Hirakubo (HRK), Shimoji (SMJ), Ohgata Risyo (OGT), Kuroshima (KRS), Kayama (KYM), Taketomi (TKT), A-sa-pi- (ASP).

In previous studies, we successfully developed/adapted highly variable DNA markers (microsatellites) for *A. digitifera* [27] and applied these markers to a small-scale (∼25 km) population genetic analysis of *A. digitifera* [28]. In this study, to clarify how *A. digitifera* maintains populations at the northern limit of its distribution, we used a population genetic approach using microsatellite markers based on a large sample size (total 602 colonies) from across a wide geographic area (∼1,000 km) covering most of the Nansei Islands (Tanega-shima, which is in a temperate area and Amami, Okinawa, Kerama, Miyako, Ishigaki, and Sekisei Reef, which are subtropical) to examine genetic connectivity and the relationship between genetic diversity and latitude (Figure 1).

## Results

### Extent of Genetic Diversity

The mean number of alleles was 5.7–11.3 for six loci at every site (average 9.97 per site). The mean heterozygosity value of all loci was 0.508–0.687 at every site, and the total mean value ± standard error (SE) was 0.592±0.012 for all sites (Table 1). No significant correlation was detected between heterozygosity and latitude (p = 0.13, Pearson's correlation coefficient). Private alleles (*PVA*) were found at some sites: in particular, two private alleles were detected at the SNS site in the Kerama region and three were at the KRS site in the Sekisei Reef region (Table 1). Departures of population heterozygosity from Hardy-Weinberg equilibrium (HWE) were suggested by *F*_IS_, which ranged from 0.026 to 0.205 for all sites (Table 1). *F*_IS_ values for MS166 and MS181 were relatively high compared to those for other loci. We excluded MS166 loci from subsequent analyses because many null alleles were suggested from the result of MICROCHECKER (see Materials and Methods, and Table S1). The ratio of the number of observed genotypes (*Ng*) to the number of individuals sampled (*N*) ranged from 0.91 to 1.00 (Table 2). The values of *Ng*/*N* were high, regardless of geographic position.

**Table 1:** The number of analyzed colonies (*N*), the number of alleles (*Na*), observed (*Ho*), expected (*He*) heterozygotes, inbreeding coefficients (*F*_IS_), and the number of private alleles (*PVA*) for each locus and site of *Acropora digitifera* at 19 sites.

		Sampling sites																			
Locus		SMY	KSN	NON	MEY	OHD	MED	UKA	MJN	SNS	IRB	YSN	UGN	HRK	SMJ	OGT	KRS	TKT	KYM	ASP	Total
	*N*	11	36	41	39	30	25	30	22	40	25	37	22	31	39	40	40	32	31	31	602
MS166*	*Na*	4	11	11	9	9	8	11	7	9	6	8	6	7	9	7	10	10	8	8	
	*Ho*	0.273	0.389	0.537	0.410	0.467	0.600	0.467	0.455	0.375	0.200	0.297	0.364	0.258	0.282	0.375	0.425	0.250	0.323	0.226	
	*He*	0.380	0.755	0.708	0.540	0.553	0.612	0.660	0.595	0.559	0.509	0.629	0.389	0.342	0.469	0.442	0.609	0.491	0.430	0.503	
	*F*_IS_	0.326	*0.496*	*0.253*	*0.252*	0.172	0.040	*0.308*	0.258	*0.341*	*0.620*	*0.537*	0.089	0.260	*0.410*	0.164	*0.313*	*0.503*	0.265	*0.562*	
	*PVA*		1											1							
MS181	*Na*	8	19	16	16	15	18	16	15	20	15	19	14	17	18	17	19	19	16	18	
	*Ho*	0.727	0.833	0.878	0.846	0.700	0.840	0.833	0.727	0.700	0.840	0.622	0.682	0.548	0.667	0.675	0.625	0.688	0.710	0.677	
	*He*	0.802	0.895	0.881	0.904	0.836	0.894	0.911	0.877	0.893	0.908	0.885	0.868	0.904	0.884	0.889	0.915	0.870	0.888	0.866	
	*F*_IS_	0.140	0.083	0.016	0.077	*0.179*	0.081	0.102	*0.193*	*0.228*	0.095	*0.310*	*0.236*	*0.407*	*0.258*	*0.252*	*0.329*	*0.225*	*0.216*	*0.233*	
	*PVA*									1					1		1				
MS182	*Na*	8	19	21	18	20	20	16	18	19	18	23	16	17	20	18	22	17	17	18	
	*Ho*	0.727	0.806	0.902	0.769	0.967	0.840	0.800	0.864	0.950	0.800	0.838	0.818	0.871	0.821	0.800	0.750	0.781	0.839	0.774	
	*He*	0.785	0.927	0.917	0.911	0.928	0.932	0.902	0.915	0.925	0.922	0.919	0.894	0.904	0.911	0.908	0.919	0.889	0.910	0.906	
	*F*_IS_	0.121	*0.145*	0.029	*0.168*	−0.025	0.119	0.129	0.080	−0.014	*0.153*	0.101	0.107	0.053	0.112	*0.132*	*0.196*	0.137	0.095	*0.161*	
	*PVA*						1			1							1				
MS8	*Na*	2	3	2	2	2	2	2	2	2	4	2	2	3	3	3	2	2	3	2	
	*Ho*	0.545	0.667	0.390	0.513	0.233	0.520	0.500	0.500	0.475	0.320	0.486	0.273	0.290	0.462	0.450	0.250	0.469	0.452	0.484	
	*He*	0.463	0.506	0.393	0.460	0.255	0.487	0.455	0.474	0.492	0.431	0.482	0.351	0.338	0.453	0.405	0.320	0.460	0.406	0.425	
	*F*_IS_	−0.132	−0.305	0.018	−0.101	0.102	−0.047	−0.082	−0.031	0.048	0.277	0.005	0.246	0.156	−0.007	−0.098	0.231	−0.002	−0.095	−0.122	
	*PVA*										1										
A.mill2-8	*Na*	2	3	3	3	3	3	3	3	3	3	3	3	3	3	3	4	3	3	3	
	*Ho*	0.455	0.250	0.463	0.308	0.367	0.400	0.567	0.455	0.450	0.320	0.378	0.091	0.419	0.282	0.400	0.325	0.281	0.484	0.290	
	*He*	0.351	0.344	0.421	0.308	0.338	0.441	0.423	0.368	0.404	0.381	0.380	0.088	0.338	0.315	0.366	0.282	0.310	0.380	0.343	
	*F*_IS_	−0.250	0.286	−0.087	0.013	−0.069	0.113	−0.325	−0.214	−0.101	0.179	0.018	−0.012	−0.226	0.118	−0.080	−0.139	0.109	−0.257	0.169	
	*PVA*																1				
A.mill2-22	*Na*	10	11	14	14	11	9	10	10	14	11	13	9	11	13	11	10	9	13	13	
	*Ho*	0.818	0.806	0.951	0.923	0.800	0.800	0.733	0.909	0.850	0.760	0.946	0.818	0.774	0.744	0.700	0.825	0.781	0.871	0.871	
	*He*	0.864	0.864	0.860	0.860	0.849	0.845	0.843	0.872	0.869	0.824	0.850	0.823	0.847	0.834	0.812	0.858	0.851	0.857	0.862	
	*F*_IS_	0.100	0.082	−0.094	−0.060	0.074	0.073	0.146	−0.019	0.034	0.098	−0.099	0.030	0.102	0.121	0.150	0.051	0.098	0.000	0.006	
	*PVA*																				
																					**mean (± SE)**
All	*Na*	5.7	11.0	11.2	10.3	10.0	10.0	9.7	9.2	11.2	9.5	11.3	8.3	9.7	11.0	9.8	11.2	10.0	10.0	10.3	9.97
	*Ho*	0.591	0.625	0.687	0.628	0.589	0.667	0.650	0.652	0.633	0.540	0.595	0.508	0.527	0.543	0.567	0.533	0.542	0.613	0.554	0.592±0.012
	*He*	0.607	0.715	0.697	0.664	0.626	0.702	0.699	0.684	0.690	0.663	0.691	0.569	0.612	0.644	0.637	0.651	0.645	0.645	0.651	0.657±0.009
	*F*_IS_	0.075	*0.140*	0.026	*0.067*	*0.077*	0.070	*0.087*	0.070	*0.095*	*0.205*	*0.153*	*0.131*	*0.155*	*0.170*	*0.123*	*0.192*	*0.176*	*0.066*	*0.165*	
	*PVA*		1				1			2	1			1	1		3				
																					

*F*_IS_ values in italics indicate significant deviations from Hardy-Weinberg equilibrium at p<0.05 after FDR correction following [44].

*We excluded MS166 from subsequent analyses because alleles of many individuals were adjusted by MICROCHECKER (Ver. 2.2.3; [30]) due to null alleles (over 20%; see Table S1).

**Table 2:** Estimates of the contribution of asexual reproduction of *Acropora digitifera* at the 19 sites.

Sampling Site	*N*	*Ng*	*Ng*/*N*
SMY	11	11	1.00
KSN	36	36	1.00
NON	41	41	1.00
MEY	39	39	1.00
OHD	30	30	1.00
MED	25	25	1.00
UKA	30	30	1.00
MJN	22	22	1.00
SNS	40	39	0.98
IRB	25	25	1.00
YSN	37	37	1.00
UGN	22	22	1.00
HRK	31	30	0.97
SMJ	39	38	0.97
OGT	40	40	1.00
KRS	40	40	1.00
TKT	32	29	0.91
KYM	31	30	0.97
ASP	31	29	0.94
mean	31.68	31.21	0.990±0.006 (± SE)

*Ng*: number of unique multilocus genotypes, *Ng*/*N*: genotypic richness.

### Population Structure

The analysis of molecular variance (AMOVA) [29] gave estimated variance values of 0.013 (∼1%) among populations and 1.737 (∼99%) within populations (total value: 1.750), and no significant difference was observed among populations (p>0.05). This indicates high levels of genetic connectivity among *A. digitifera* populations in this area, which is also supported by the small values of pairwise *F*_ST_ (≤0.033). Significant differences were detected between some sites (Table 3), but *F*_ST_ values were not always low between geographically close sites. Some pairwise *F*_ST_ values generated between the OHD site in the Okinawa region and sites in other regions were significantly different from zero (6 cases). Significant differentiations were also detected between SNS site in the Kerama region and some sites in the Ishigaki, Sekisei Reef regions. Also, pairwise *F*_ST_ values between some sites in the southern part of the Nansei Islands (Miyako, Ishigaki, and Sekisei Reef) and KSN site in the Amami region tended to be significantly different from zero. Despite the short geographic distance between them, significant genetic differentiations were observed between IRB site in the Miyako region and UGN site in the Ishigaki region, between sites in the Miyako region and those in the Sekisei Reef region. However, the sites in the Miyako region were genetically closer to some sites in the Amami, Okinawa, and Kerama regions. A similar pattern showing no correlation between geographic and genetic distances was found between the Sekisei Reef region and other regions. For example, no significant differences were found between some sites in the Sekisei Reef region and those in the Tanega-shima, Okinawa, Kerama regions. These results did not change after adjusting for the genotypes using MICROCHECKER [30], although some of the pairwise *F*_ST_ values were changed slightly (Table S2).

**Table 3:** *Acropora digitifera* pairwise population *F*_ST_ via AMOVA values estimated among sites in the Nansei Islands.

Region	T	A	A	A	O	O	O	K	K	M	M	I	I	S	S	S	S	S	S
Site	SMY	KSN	NON	MEY	OHD	MED	UKA	MJN	SNS	IRB	YSN	UGN	HRK	SMJ	OGT	KRS	TKT	KYM	ASP
SMY																			
KSN	0.011																		
NON	0.006	0.006																	
MEY	0.012	0.003	0.002																
OHD	*0.033*	*0.022*	0.007	0.015															
MED	0.000	−0.006	−0.001	0.003	*0.022*														
UKA	0.001	0.002	−0.004	−0.001	0.017	−0.007													
MJN	0.023	−0.001	0.002	−0.002	0.017	−0.005	0.000												
SNS	0.014	0.002	0.007	−0.001	*0.024*	−0.006	0.001	−0.006											
IRB	0.020	*0.018*	0.008	0.010	*0.018*	0.010	0.010	0.010	0.014										
YSN	0.012	−0.001	0.003	0.000	*0.019*	−0.005	0.000	0.003	0.001	0.015									
UGN	0.023	0.017	0.013	0.003	0.008	0.021	0.011	0.015	*0.023*	*0.023*	0.013								
HRK	0.014	*0.020*	0.007	0.011	0.009	0.012	0.003	0.014	*0.018*	0.015	0.015	0.007							
SMJ	0.006	0.006	0.002	−0.002	0.013	0.001	0.000	0.009	0.007	0.010	−0.002	0.002	0.008						
OGT	0.019	*0.016*	0.010	0.010	0.008	0.010	0.006	0.008	*0.015*	*0.019*	*0.016*	0.017	0.009	*0.014*					
KRS	*0.029*	*0.018*	0.005	0.005	0.006	0.016	0.011	0.009	*0.019*	0.006	*0.015*	0.007	0.009	0.006	0.010				
TKT	0.002	0.008	0.011	0.008	0.016	0.002	0.003	0.009	0.006	0.010	0.008	0.018	0.013	0.004	0.005	*0.016*			
KYM	0.013	0.009	0.002	0.005	0.004	0.000	−0.001	0.008	0.007	0.005	0.002	0.007	0.003	−0.003	0.002	0.001	0.001		
ASP	0.005	0.010	0.002	−0.001	0.010	0.001	0.004	0.004	0.002	0.005	−0.001	0.007	0.008	−0.006	0.011	0.001	0.005	−0.004	

Statistical significance was calculated, and probability values based on 999 permutations are shown. Statistical significance levels for all pairwise tests were p<0.05 after adjusting for multiple comparisons using a FDR correction following [44]. Values in italics are significant. A letter in regions suggests the first letter of sampling region; T: Tanega-shima, A: Amami, O: Okinawa, K: Kerama, M: Miyako, I: Ishigaki, S: Sekisei Reef. Furthermore, pairwise *F*_ST_ via AMOVA were also calculated from the allele data adjusted by MICROCHECKER (Ver. 2.2.3; [30]) (Table S2).

**Figure 2:**
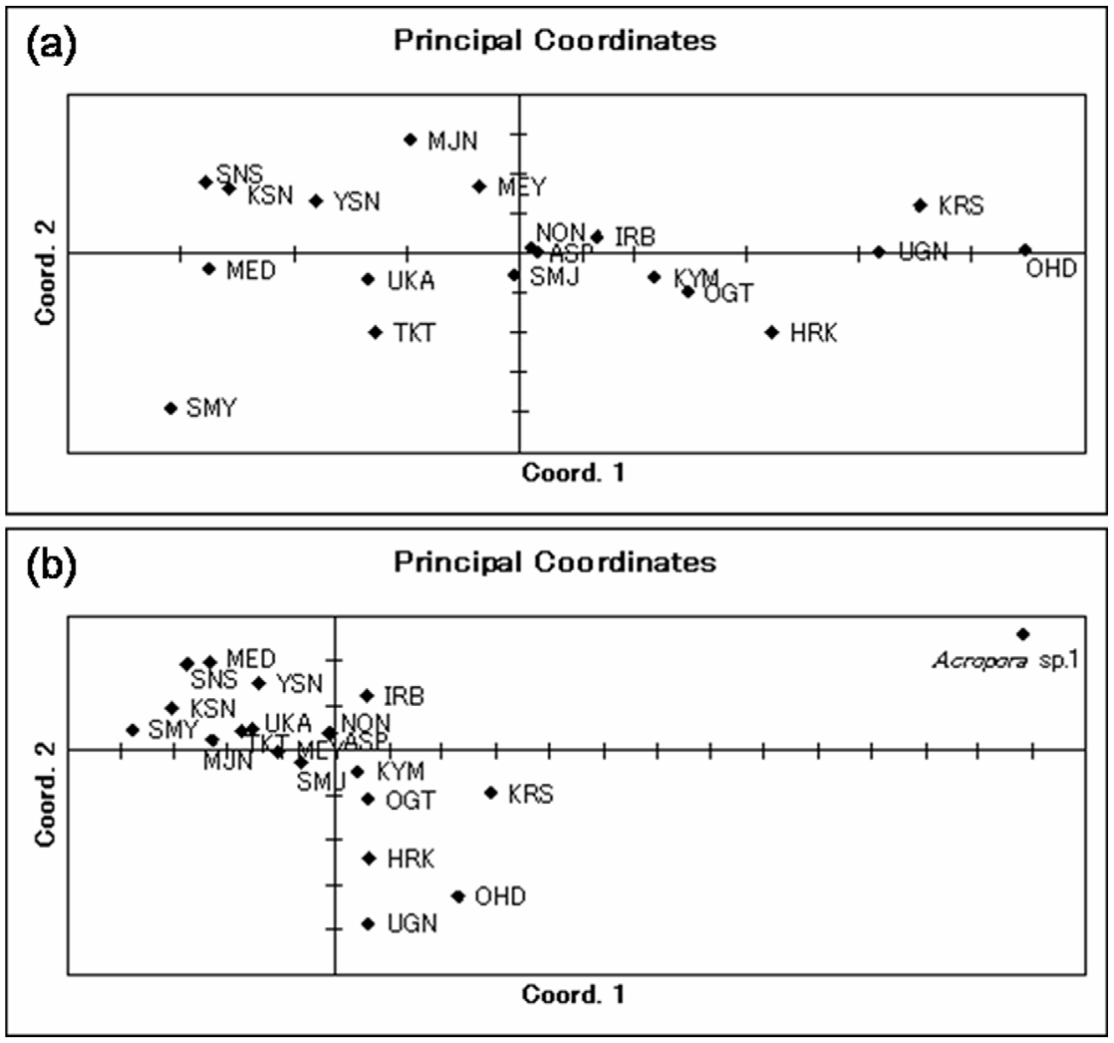
Plots of the principal coordinate analysis (PCA) from the covariance matrix with data standardization calculated using GenAlEx for *Acropora digitifera*. (a): Plots of *A. digitifera* at 19 sites. *F*_ST_: the first two axes explain 58.43% of the variation (the first axis explains 38.99%, the second axis 19.44% of variation). The minimum scale is 0.01 values on the *x* and *y*-axis. (b): Plots of *A. digitifera* including the data of *Acropora* sp. 1. *F*_ST_: the first two axes explain 70.30% of the variation (the first axis explains 57.12%, the second axis 13.18% of variation). The minimum scale is 0.01 values on the *x* and *y*-axis.

Principal coordinate analysis (PCA; [31]) revealed high degrees of genetic differentiation among sites within the same region (Figure 2), even though little differentiation occurred among regions. In the analysis of *A. digitifera* at 19 sites (Figure 2a), the first two axes explained 58.43% of the variation (the first axis 38.99%, and the second axis 19.44%). The OHD plot was remote from the plots of the other two Okinawa region sites (MED and UKA) in the PCA graph. However, the MED and UKA plots were near plots of sites from the Amami, Kerama, and Miyako regions. In the analysis including *Acropora* sp.1 (cryptic species of *A. digitifera* [32] as an outgroup (Figure 2b), the first two axes explained 70.30% of the variation (the first axis 57.12%, and the second axis 13.18%), and all plots derived from *A. digitifera* are gathering compared with the location of plot derived from *Acropora* sp. 1, but some plots (e.g. OHD) from *A. digitifera* are remote from other plots. The results of PCA did not change after adjusting for the genotypes using MICROCHECKER (data not shown). The result of STRUCTURE [33] analysis also indicated that there was no subdivision of populations indicating that the degree of genetic differentiation was markedly low. This tendency was confirmed when performing estimations of population structure for other values of *K*, i.e., *K* = 3 to 7. Also, the estimated log probability of the data, LnP(D) gradually decreased with increases in *K* value, *K* = 2: LnP(2) = −11396.3, *K* = 3: LnP(3) = −12003.0, *K* = 4: LnP(4) = −12668.2, *K* = 5: LnP(5) = −12313.1, *K* = 6: LnP(6) = −12141.3, *K* = 7: LnP(7) = −12223.5.

## Discussion

In our study, the existence of genetic connectivity among *A. digitifera* populations over the large geographic area (∼1,000 km) of the Nansei Islands was confirmed. *F*_ST_ values for *A. digitifera* were 0.033 and smaller for all site combinations (Table 3). These values mean that among *A. digitifera* in the Nansei Islands, the level of genetic differentiation was low and the degree of connectivity was high, regardless of geographic distance. Our result is in contrast with some previous studies that showed the existence of strong genetic subdivision in some spawning corals. Baums et al. [19] and Underwood [22] showed clear genetic differentiations in *Acropora* species which were thought to be related to physical factors limiting larval dispersal. In a high-latitude region in East Africa, *Pocillopora verrucosa* showed strong genetic differentiation across a distance of about 1,000 km, a scale almost the same as that of this study [21]. Furthermore, in the present study, no tendency was observed for private alleles to be found at sites where significant genetic differentiation was detected compared to other sites. For example, although analyzed colonies from KRS in the Sekisei Reef region contained three private alleles, OHD in the Okinawa region and UGN in the Ishigaki region had no private alleles, although these populations showed significant genetic differentiation from some other populations. Therefore, populations of *A. digitifera* in the Nansei Islands appear to have complex and various connective patterns. This result is also supported by the STRUCTURE analysis showing that genetic division did not occur. In this study, we could find no evidence for population structure at all sites with five microsatellite loci. The high values of *Ng*/*N* (mean ± SE of *Ng*/*N* was 0.990±0.006) suggest that *A. digitifera* colonies in the Nansei Islands propagate mainly through sexual reproduction. Thus, the wide range of genetic connectivity among *A. digitifera* populations in the Nansei Islands is believed to be maintained by the dispersal of sexually produced planula larvae, and not by asexual reproduction.

While the degree of the genetic connectivity of *A. digitifera* was high in the Nansei Islands, significant genetic differentiation between close sites in some regions were confirmed in some cases (e.g., OHD-MED within the Okinawa region). In addition, PCA indicated that the *F*_ST_ values of this species varied considerably among sites within a region. Although one must note that low *F*_ST_ values may be easily detected when using highly variable markers such as microsatellites [34], Hedrick [35] mentioned that this potential problem is not common in traditional markers such as allozymes or other loci with low variances. In another *A. digitifera* population genetic study using allozymes, Nishikawa et al. [36] also suggested disagreement between genetic connectivity and geographic distance in *A. digitifera* in the Ryukyu archipelago. Therefore, our result appears to have been caused by ecological or biological factors rather than by the characteristics of the markers. Physical and topological factors may affect the movement and habitat selection of this species, causing local genetic differentiation. Miller and Ayre [37] showed that the degree of genetic differentiation was not correlated with geographic distance in populations of *Pocillopora damicornis* on Lord Howe Island, and they found that *F*_ST_ values throughout the North Bay and other sites in a protected lagoon (several kilometers away) were lower than at sites in the Old Gulch (only 1 km away) located in the open sea. They suggested that the existence of a shallow reef interfered with genetic exchanges between the close sites. Such topographic effects may also be present around Okinawa Island because the coastal topography of the Nansei Islands is complex. Many gulfs exist along the coast of Okinawa Island, and the shapes of reefs around the Nansei Islands vary greatly, which would influence larval recruitment. For example, in contrast to the geographic distances between them, a population of *Goniastrea aspera* at Sesoko on the west coast of Okinawa Island was genetically closer to a population in the Kerama Islands located west of the Okinawa Islands than to a population at OHD on the southeast coast of Okinawa Island [38]. The same situation would likely apply to the gene flow of *A. digitifera* observed in our results. In addition, differences in the environmental factors (e.g., temperature, turbidity) affecting larval recruitment and the survival of adult corals may also be related to the local genetic differentiation observed in this study because environmental conditions around the Nansei Islands vary greatly among sites [26]. Environmental conditions in coral habitats are not necessarily identical at all sites, which may possibly cause differences in larval recruitment patterns and the survival of recruited corals, leading to local genetic differentiation.

We found no significant relationship between genetic diversity and latitude in *A. digitifera*. To date, some studies of marine animals have shown that genetic diversity decreases at the boundaries of geographic distribution [21], [39], [40]. For example, the endangered tideland snail, *Batillaria zonalis* has low mitochondrial genetic diversity at the northern and southern edges of its geographic distribution range in Japan [40]. This low genetic diversity might be attributable to population bottlenecks due to historical environmental variations and/or the recent foundation of populations in the marginal areas of its inhabitable range. Also, in the eelgrass *Zostera marina*, the numbers of leaf shoots, dry biomass, and faunal abundance were found to increase according to the increase in microsatellite genotypes, despite near-lethal sea temperatures, and declines in genotypic diversity were related to decreases of leaf shoots, dry biomass and faunal abundance [41]. Coral populations of five species on high-latitude reefs at Lord Howe Island off the east coast of Australia showed lower levels of genetic diversity compared to populations on the Great Barrier Reef [39]. In case of *Acropora tenuis* in the northwest of Australia, significant differences in genetic diversity were detected between inshore and offshore regions, with lower diversity observed on higher-latitude reefs [22]. Decreased genetic diversity may mean decreased potential for adapting to environmental changes [39]. In contrast, we found no tendency for the average number of alleles or the heterozygosity of *A. digitifera* populations to decrease with latitude. Decreased heterozygosity was not detected in the Tanega-shima region, which is almost the northern limit of *A. digitifera*'s geographic distribution, even though the sample size was small (*N* = 11). The exchange of larvae creates and maintains high levels of genetic diversity, which is crucial in terms of resilience against disturbance [4]. Migrants may carry new alleles that are integrated into populations through dispersal, creating new gene combinations on which selection can act [4]. The spread of selectively advantageous alleles at DNA loci involved in physiological responses, such as resistance to bleaching, is another potential consequence of migration [4]. Considering the high level of gene flow and the maintenance of high genetic diversity among *A. digitifera* populations across a large geographic area, even if mass bleaching occurs, *A. digitifera* populations might be able to show rapid numerical recovery, even in temperate regions. A possibility exists that *A. digitifera* populations in the Nanei Islands remain relatively tolerant to environmental changes such as sudden elevations in sea temperature by sharing a variety of alleles. This potential would have been maintained by strong gene flow caused by the Kuroshio Current [13].

In conclusion, our results show that regardless of the latitude of their habitat, *A. digitifera* populations in the Nansei Islands seem to have high recovery potential even after high levels of disturbance, provided that populations on unaffected reefs are maintained. However, note that we examined only one species and that these results should not be simply applied to other coral species, including other *Acropora* spp. Therefore, comparative analyses of genetic connectivity in several coral species, including both spawners and brooders, should be performed using highly variable markers such as microsatellites to enhance the management of coral communities in our study area based on a more generalized view of coral populations at the periphery of their geographical distribution. Also, if connections and recruitment from outside decrease, peripheral populations are expected to exhibit reduced levels of genetic and genotypic diversity due to the combined effects of bottlenecks, inbreeding, and site-specific selection [37]. Thus, the present results may not always apply to peripheral populations of this species distributed in higher latitude areas in Japan. Future monitoring of the population and genetic dynamics of this species is necessary.

## Materials and Methods

### Sampling

We established seven geographic sampling regions (Tanega-shima, Amami, Okinawa, Kerama, Miyako, Ishigaki, and Sekisei Reef) in the Nansei Islands (Figure 1). All samples were collected in strict accordance with good animal practice as defined by the relevant national and/or local animal welfare bodies, and all sampling requiring permission for this study within Okinawa Prefecture was approved by the prefecture. Fragments of *A. digitifera* were taken at each sampling site from haphazardly selected colonies that were at least 3 m apart. Coral fragments were preserved in 100% ethanol in 1.5-ml Eppendorf tubes and were then transported to the laboratory.

### Genomic DNA Extraction

Using an AquaPure Genomic DNA kit (Bio-Rad, Hercules, CA, USA), genomic DNA was extracted from the surface tissues of *A. digitifera* fragments by removing the coral skeleton from sample tubes containing coral tissues. We usually extracted genomic DNA from the surface tissues as above, but in the case of samples from Sekisei Reef, genomic DNA was extracted not only from the surface tissues but also from skeletons. The skeletons were washed in 100% ethanol, dried, crushed with a pestle, and suspended in 400 µl SE buffer (5 M NaCl 1.5%, 0.5 M EDTA 5%, SDS 0.5%) in a 1.5-ml Eppendorf tube. We then added 3 µl Proteinase K (20 mg ml^−1^) and incubated samples at 37°C to 50°C for 24 to 72 hours. After that, we added 160 µl 5 M NaCl and 530 µl chloroform and vigorously mixed the samples using a vortex mixer before centrifuging. We then took the water layer and carried out standard ethanol precipitation.

### Genotyping

We used microsatellite marker developed for *A. digitifera* by Nakajima et al. [27] (Table 4). We also adapted primers developed for *Acropora palmata* by Baums et al. [16] and *Acropora millepora* by van Oppen et al. [18] which can work in *A. digitifera* (Table 4). We amplified DNA using the multiplex PCR method (adding two primer sets to one PCR) using Ex Taq DNA polymerase (Takara, Tokyo, Japan) with 10× Ex Taq buffer, 4 pM dNTPs (1 pM each), 100 nM primers (for 2 loci; Table 4), 0.125 U Ex Taq DNA polymerase, generally <5 ng µl^−1^ (multiC: <1 ng µl^−1^) template DNA, and MilliQ water (Millipore, Billerica, MA, USA) for a total reaction volume of 5 µl. Amplifications were carried out in a PC-818 touchdown thermocycler (Astec, Chattanooga, TN, USA) operated under the following conditions: 95°C for 5 minutes, followed by 35 cycles at 95°C for 30 seconds, 50°C (gradient: −0.1°C cycle^−1^) for 30 seconds, 72°C for 1 minute, and a final 72°C extension for 30 minutes. Allelic variations were analyzed using a DNA capillary sequencer (CEQ-8800; Beckman Coulter, Fullerton, CA, USA). When alleles were unclear or not detected, normal PCR (i.e., not multiplex) was conducted under the 100 nM primers for 1 locus (forward and reverse were 50nM, respectively). We did not find shifts in allele size between multiplex and normal PCR. In Sekisei Reef samples, when we could not detect clear genotypes using DNA from tissues of *A. digitifera*, we also used DNA extracted from the skeleton (∼50–300 ng µl^−1^) as a template. For regular PCR with the Sekisei Reef samples, we diluted skeletal DNA (up to 10 times) to obtain the appropriate DNA concentration, if required.

**Table 4:** Microsatellite markers of *Acropora* species used in this study.

Locus	Repeat motif	Primer sequence (5′-3′)	Size range of alleles (nt)	Multiplex and primers concentration (nM)	Reference
MS166	(AAT)_2_AAAAATAAC(AAT)_4_	D3 (green)-TCTACCCGCAATTTTCATCA	116–160	multiA	Baums et al. [16]
		CGCTCTCCTATGTTCGATTG		40 (F: 20, R: 20)	
MS181	(AAT)_5_GAT(AAT)_5_ATT(AAT)_3_	D4 (blue)-TTCTCCACATGCAAACAAACA	143–269	multiA	Baums et al. [16]
		GCCAGGATAGCGGATAATGA		60 (F: 30; R: 30)	
MS182	(AAT)_10_	D4 (blue)-TCCCACAACTCACACTCTGC	128–231	multiB	Baums et al. [16]
		ACGCGGAAATAGTGATGCTC		46 (F: 23; R: 23)	
MS8	(CT)_3_GT(CT)_5_	D3 (green)-GATCCGTCACACTTGTTCTAAGG	80–91	multiB	Nakajima et al. [27]
		TGACTGTCAGAGTAGAGGGAAGG		54 (F: 27; R: 27)	
A.mill2-8	(AC)_6_	D2 (black)- AGGTTTCTATGGGAACGTCG	90–96	multiC	van Oppen et al. [18]
		TGAACTTCAAGTAATTTTGCCAG		50 (F: 25; R: 25)	
A.mill2-22	(AC)_10_	D4 (blue)-CTGTGGCCTTGTTAGATAGC	158–192	multiC	van Oppen et al. [18]
		AGATTTGTGTTGTCCTGCTT		50 (F: 25; R: 25)	

D2 (black), D3 (green), and D4 (blue) in the primer sequence are fluorescent dye labels (Sigma-Genosys, St. Louis, MO, USA). The size range was suggested by analysis using a DNA sequencer (CEQ-8800; Beckman Coulter, Fullerton, CA, USA). Two capitals in the column multiplex and primers concentration represent forward (F) and reverse (R), respectively.

### Statistical Analyses

The numbers of alleles, allele frequencies, and the number of private alleles were calculated using the GenAlEx program (Ver. 6.2) [42]. Also, we used MICROCHECKER (Ver. 2.2.3; [30]) with Oosterhout algorithm to examine the influence of undetectable alleles in microsatellite loci. In MS166 locus, the existence of many null alleles were confirmed (over 20% of all 602 individuals changed the allele pattern to heterozygosis from homozygosis after the adjustment by MICROCHECKER; Table S1). Therefore, we excluded MS166 from all subsequent analyses; calculation of *Ng* and *Ng/N* values, pairwise AMOVA *F*_ST_ values, principal coordinate analysis (PCA; [31]), estimating subdivision of populations through the STRUCTURE [33] analysis. *F*_IS_ values were also calculated using FSTAT version 2.9.3.2 [43] because gaps between observed heterozygosity (*Ho*) and expected heterozygosity (*He*) under HWE are in proportion to the values of the inbreeding coefficient (*F*_IS_), that is, positive and negative *F*_IS_ values suggest deficits and excesses of heterozygotes, respectively. The exact test for departure from HWE was also performed using FSTAT. Significance levels were adjusted using a false discovery rate (FDR) correction following [44].

The extent of asexual reproduction was estimated from the genotypic diversity of each population. If several unique multilocus genotypes were detected, *Ng* represents an estimate of the minimum number of clones present in a population. When *N* indicates the number of collected and genotyped individual colonies, *Ng*/*N* provides an index of the effects of asexual reproduction and suggests genotypic richness [45]. When *Ng*/*N* = 1, all of the collected colonies in a population are unique (no clones); *Ng*/*N* approaches zero when a population has only a single genotype (all clones).

To measure the proportion of genetic variation between sites, we used *F*-statistics via AMOVA. This analysis was carried out using GenAlEx (Ver. 6.2) [42] to test the significance of all estimates based on 999 random permutations. A low pairwise *F*_ST_ indicates a high extent of gene flow and vice versa. Significance levels were adjusted using FDR corrections. Adjusted allele data by MICROCHECKER (Ver. 2.2.3; [30]) were used for the calculation of pairwise *F*_ST_ values. Furthermore, we constructed a principal coordinate analysis (PCA; [31]) graph in GenAlEx to visualize a covariance matrix with data standardization derived from the pairwise *F*_ST_ values to more effectively view the patterns of genetic distance among populations. PCA generates a set of rectangular axes for which each successive dimension maximizes the remaining variance in the data. Patterns revealed by the first two principal coordinate axes were found to be representative of higher-order axes, and thus only the first two dimensions were plotted. Furthermore, we added the data of *Acropora* sp. 1 (cryptic species of *A. digitifera* [32], which was referred from Nakajima et al. [27]) as an outgroup.

### Estimating the Subdivision of Populations

Population structure was inferred from microsatellite data using STRUCTURE software (Ver. 2.2) [33]. This software applies a Bayesian clustering approach to identify populations possessing a characteristic set of allele polymorphisms based on genotyping data from microsatellite alleles. A burn-in period of 100,000 followed by 1,000,000 Markov chain Monte Carlo (MCMC) replications was used for population clustering. We performed this analysis by assuming values of *K* from 2 to 7 with regard to the number of sampling regions. The values of *K* show the number of potential clusters.

## Supporting Information

Table S1 The number and rates of individuals changed the allele pattern to heterozygosis from homozygosis after the adjustment by MICROCHECKER (Ver. 2.2.3; [30]) for each locus and site of *Acropora digitifera* at 19 sites. N is the number of analyzed colonies. *ADJ* and % suggest the number and rate of individuals adjusted by MICROCHECKER, respectively. *We excluded MS166 from subsequent analyses because alleles of many individuals were adjusted due to null alleles (over 20%).
(0.08 MB DOC)

Table S2 *Acropora digitifera* pairwise population *F*_ST_ via AMOVA values estimated among sites in the Nansei Islands and adjusted by MICROCHECKER (Ver. 2.2.3; [30]). Statistical significance was calculated, and probability values based on 999 permutations are shown. Statistical significance levels for all pairwise tests were p<0.05 after adjusting for multiple comparisons using a FDR correction following [44]. Values in italics are significant. A letter in regions suggests the first letter of sampling region; T: Tanega-shima, A: Amami, O: Okinawa, K: Kerama, M: Miyako, I: Ishigaki, S: Sekisei Reef.
(0.09 MB DOC)
