# Supplementary material for: Gene Flow and Genetic Diversity of a Broadcast-Spawning Coral in Northern Peripheral Populations
Source: PLoS One. 2010 Jun 16;5(6):e11149. doi: 10.1371/journal.pone.0011149 (PMC2886843; doi:10.1371/journal.pone.0011149)
Supplement: Table S1 — The number and rates of individuals changed the allele pattern to heterozygosis from homozygosis after the adjustment by MICROCHECKER (Ver. 2.2.3; [30]) for each locus and site of Acropora digitifera at 19 sites. N is the number of analyzed colonies. ADJ and % suggest the number and rate of individuals adjusted by MICROCHECKER, respectively. *We excluded MS166 from subsequent analyses because alleles of many individuals were adjusted due to null alleles (over 20%). (0.08 MB DOC) [file pone.0011149.s001.doc]

|  |  | **Sampling sites** | | | | | | | | | | | | | | | | | | |  |
| --- | --- | --- | --- | --- | --- | --- | --- | --- | --- | --- | --- | --- | --- | --- | --- | --- | --- | --- | --- | --- | --- |
| **Locus** |  | **SMY** | **KSN** | **NON** | **MEY** | **OHD** | **MED** | **UKA** | **MJN** | **SNS** | **IRB** | **YSN** | **UGN** | **HRK** | **SMJ** | **OGT** | **KRS** | **TKT** | **KYM** | **ASP** | **Total** |
|  | *N* | 11 | 36 | 41 | 39 | 30 | 25 | 30 | 22 | 40 | 25 | 37 | 22 | 31 | 39 | 40 | 40 | 32 | 31 | 31 | 602 |
| MS166* | *ADJ* |  | 14 | 6 | 5 |  |  | 6 | 3 | 9 | 11 | 17 |  | 5 | 11 |  | 10 | 11 | 7 | 11 | 126 |
|  | % |  | 38.89 | 14.63 | 12.82 |  |  | 20.00 | 13.64 | 22.50 | 44.00 | 45.95 |  | 16.13 | 28.21 |  | 25.00 | 34.38 | 22.58 | 35.48 | 20.93 |
| MS181 | *ADJ* |  |  |  |  | 2 |  |  |  | 5 |  | 6 |  | 6 | 6 | 3 | 4 | 3 | 1 | 4 | 40 |
|  | % |  |  |  |  | 6.67 |  |  |  | 12.50 |  | 16.22 |  | 19.35 | 15.38 | 7.50 | 10.00 | 9.38 | 3.26 | 12.90 | 6.64 |
| MS182 | *ADJ* |  |  |  | 1 |  |  |  |  |  |  |  |  |  |  |  | 1 |  |  |  | 2 |
|  | % |  |  |  | 2.56 |  |  |  |  |  |  |  |  |  |  |  | 2.50 |  |  |  | 0.00 |
| MS8 | *ADJ* |  |  |  |  |  |  |  |  |  |  |  |  |  |  |  |  |  |  |  | 0 |
|  | % |  |  |  |  |  |  |  |  |  |  |  |  |  |  |  |  |  |  |  | 0.00 |
| A.mill2-8 | *ADJ* |  |  |  |  |  |  |  |  |  |  |  |  |  |  |  |  |  |  |  | 0 |
|  | % |  |  |  |  |  |  |  |  |  |  |  |  |  |  |  |  |  |  |  | 0.00 |
| A.mill2-22 | *ADJ* |  |  |  |  |  |  |  |  |  |  |  |  |  |  |  |  |  |  |  | 0 |
|  | % |  |  |  |  |  |  |  |  |  |  |  |  |  |  |  |  |  |  |  | 0.00 |

**Table S1.**
