# Supplementary material for: Gene Flow and Genetic Diversity of a Broadcast-Spawning Coral in Northern Peripheral Populations
Source: PLoS One. 2010 Jun 16;5(6):e11149. doi: 10.1371/journal.pone.0011149 (PMC2886843; doi:10.1371/journal.pone.0011149)
Supplement: Table S2 — Acropora digitifera pairwise population F ST via AMOVA values estimated among sites in the Nansei Islands and adjusted by MICROCHECKER (Ver. 2.2.3; [30]). Statistical significance was calculated, and probability values based on 999 permutations are shown. Statistical significance levels for all pairwise tests were p<0.05 after adjusting for multiple comparisons using a FDR correction following [44]. Values in italics are significant. A letter in regions suggests the first letter of sampling region; T: Tanega-shima, A: Amami, O: Okinawa, K: Kerama, M: Miyako, I: Ishigaki, S: Sekisei Reef. (0.09 MB DOC) [file pone.0011149.s002.doc]

| **Region** | **T** | **A** | **A** | **A** | **O** | **O** | **O** | **K** | **K** | **M** | **M** | **I** | **I** | **S** | **S** | **S** | **S** | **S** | **S** |
| --- | --- | --- | --- | --- | --- | --- | --- | --- | --- | --- | --- | --- | --- | --- | --- | --- | --- | --- | --- |
| **Site** | **SMY** | **KSN** | **NON** | **MEY** | **OHD** | **MED** | **UKA** | **MJN** | **SNS** | **IRB** | **YSN** | **UGN** | **HRK** | **SMJ** | **OGT** | **KRS** | **TKT** | **KYM** | **ASP** |
| SMY |  |  |  |  |  |  |  |  |  |  |  |  |  |  |  |  |  |  |  |
| KSN | 0.011 |  |  |  |  |  |  |  |  |  |  |  |  |  |  |  |  |  |  |
| NON | 0.006 | 0.006 |  |  |  |  |  |  |  |  |  |  |  |  |  |  |  |  |  |
| MEY | 0.012 | 0.003 | 0.002 |  |  |  |  |  |  |  |  |  |  |  |  |  |  |  |  |
| OHD | *0.031* | *0.021* | 0.005 | 0.013 |  |  |  |  |  |  |  |  |  |  |  |  |  |  |  |
| MED | 0.000 | -0.006 | -0.001 | 0.003 | *0.020* |  |  |  |  |  |  |  |  |  |  |  |  |  |  |
| UKA | 0.001 | 0.002 | -0.004 | -0.001 | 0.014 | -0.007 |  |  |  |  |  |  |  |  |  |  |  |  |  |
| MJN | 0.023 | -0.001 | 0.002 | -0.002 | 0.016 | -0.005 | 0.000 |  |  |  |  |  |  |  |  |  |  |  |  |
| SNS | 0.015 | 0.002 | 0.008 | -0.001 | *0.023* | -0.005 | 0.001 | -0.005 |  |  |  |  |  |  |  |  |  |  |  |
| IRB | 0.020 | *0.018* | 0.008 | 0.010 | 0.017 | 0.010 | 0.010 | 0.010 | 0.013 |  |  |  |  |  |  |  |  |  |  |
| YSN | 0.014 | -0.001 | 0.003 | 0.000 | *0.018* | -0.004 | 0.000 | 0.004 | 0.000 | 0.015 |  |  |  |  |  |  |  |  |  |
| UGN | 0.023 | 0.017 | 0.013 | 0.003 | 0.007 | 0.021 | 0.011 | 0.015 | *0.024* | *0.023* | 0.014 |  |  |  |  |  |  |  |  |
| HRK | 0.015 | *0.020* | 0.008 | 0.012 | 0.007 | 0.013 | 0.004 | 0.015 | *0.016* | 0.015 | 0.013 | 0.009 |  |  |  |  |  |  |  |
| SMJ | 0.008 | 0.007 | 0.002 | -0.002 | 0.012 | 0.002 | 0.000 | 0.009 | 0.006 | 0.009 | -0.002 | 0.003 | 0.007 |  |  |  |  |  |  |
| OGT | 0.019 | *0.016* | 0.010 | 0.010 | 0.008 | 0.009 | 0.005 | 0.008 | *0.014* | *0.019* | *0.015* | 0.017 | 0.007 | *0.013* |  |  |  |  |  |
| KRS | *0.029* | *0.018* | 0.005 | 0.006 | 0.005 | 0.017 | 0.011 | 0.010 | *0.018* | 0.006 | *0.015* | 0.008 | 0.008 | 0.005 | 0.009 |  |  |  |  |
| TKT | 0.002 | 0.007 | 0.010 | 0.007 | *0.016* | 0.001 | 0.001 | 0.008 | 0.006 | 0.009 | 0.007 | 0.018 | 0.010 | 0.003 | 0.004 | *0.015* |  |  |  |
| KYM | 0.012 | 0.009 | 0.001 | 0.004 | 0.004 | 0.000 | -0.001 | 0.008 | 0.007 | 0.004 | 0.002 | 0.007 | 0.003 | -0.003 | 0.002 | 0.001 | 0.001 |  |  |
| ASP | 0.006 | 0.010 | 0.002 | -0.002 | 0.009 | 0.001 | 0.003 | 0.004 | 0.002 | 0.005 | -0.001 | 0.007 | 0.006 | -0.006 | 0.011 | 0.000 | 0.004 | -0.004 |  |

**Table S2.**
